# Systemic inflammatory markers in neuropathic pain, nerve injury and recovery

**DOI:** 10.1097/j.pain.0000000000002386

**Published:** 2022-03-01

**Authors:** Oliver Sandy-Hindmarch, David L Bennett, Akira Wiberg, Dominic Furniss, Georgios Baskozos, Annina B Schmid

**Affiliations:** 1 Nuffield Department for Clinical Neurosciences, University of Oxford, OX3 9DU, Oxford, United Kingdom; 2 Nuffield Department of Orthopaedics, Rheumatology, and Musculoskeletal Sciences, University of Oxford, OX3 7LD, Oxford, United Kingdom

## Abstract

The role that inflammation plays in human nerve injury and neuropathic pain is incompletely understood. Previous studies highlight the role of inflammation in the generation and maintenance of neuropathic pain, but the emerging evidence from the preclinical literature for its role in the resolution of neuropathic pain remains to be explored in humans. Here, we use carpal tunnel syndrome (CTS) as a human model system of nerve injury and neuropathic pain to determine changes in serum cytokine protein levels and gene expression levels before (active stage of disease) and after carpal tunnel decompression surgery (recovery). Fifty-five CTS patients were studied and 21 healthy age and gender matched participants served as controls. In the active stage of the disease (CTS before surgery vs healthy controls), *PTGES2* mRNA was decreased in patients (adjusted p=0.013), while TGF-β and CCL5 protein levels were increased (adjusted p=0.016 and p=0.047 respectively). In the resolution phase (CTS before surgery vs after surgery), *IL-9* mRNA was increased after surgery (adjusted p=0.014) and expression of *IL-6* mRNA and IL-4 protein levels were increased before surgery (adjusted p=0.034 and p=0.002 respectively). *IL-9* mRNA expression negatively correlated with several (neuropathic) pain scores. In contrast, protein levels of IL-4 positively correlated with pain scores. In conclusion, we demonstrate specific dysregulation of systemic cytokine expression both in the active and resolution phases of nerve injury and neuropathic pain. IL-9 represents an interesting candidate associated with resolution of nerve injury and neuropathic pain.

## Introduction

It is well established from preclinical models that neuroinflammation plays an important role in the initiation and maintenance of neuropathic pain [20,50]. The presence of neuroinflammation has also been confirmed in patients with severe peripheral neuropathies, where nerve biopsies are warranted [22,63,73,74,82]. However, the limited access to human neural tissue means that the clinical detection of inflammation mostly relies on indirect measures, such as the presence of systemic inflammatory markers in the blood. Most studies report changes in blood inflammatory markers in patients with neuropathic pain, however the limited number of cytokines studied (e.g., TNF, IL-6, IL1b, IL-4, IL-10) prevents a comprehensive overview and discovery of contributions of less studied cytokines in most studies [9,34,36,64,77].

Along with the role of inflammation during initiation and maintenance of neuropathic pain, its contribution to recovery and resolution has gained increasing interest in recent years [17,38]. Studying the role of the immune system in the resolution of neuropathic pain remains challenging in humans, as many neuropathic pain conditions are chronic, and treatments are often only modestly effective. There is some indication from analysis of serial blister fluid from patients with complex regional pain syndrome that inflammatory markers normalise over time, however this does not seem to be related to treatment outcome or disease characteristics [40,78]. There is also evidence of a role for inflammatory mediators in patients with sciatica, where changes in some serum inflammatory mediators over time show associations with pain and recovery [34]. However, most studies restrict their analyses to a limited number of inflammatory markers (e.g., IL-6, IL-8, IL-4, IL-1β, TNF, CRP) [37,54,55,59,67,85]. Comprehensive longitudinal analyses of human inflammatory changes at both gene and protein level are needed to shed light on the role of inflammation in neuropathic pain maintenance and resolution.

Here, we use carpal tunnel syndrome (CTS) as a human model system to prospectively study inflammation in the context of neuropathic pain and its resolution. CTS is the most common peripheral neuropathy and cause for neuropathic pain [1]. Unlike many other neuropathic pain conditions, CTS can successfully be treated by a single and time-locked intervention: surgical decompression. CTS therefore represents an ideal model system that allows the prospective evaluation of inflammation from the active stage of nerve injury (pre-surgery) to recovery (post-surgery). There is growing evidence for a role of blood inflammatory mediators in the active stage of CTS [49,69], although conflicting results have also been reported [26,36,69]. The most comprehensive cross-sectional study to date suggested that serum concentrations of CCL5, VEGF, CXCL8 and CXCL10 as well as the number of central and effector memory T-cell populations were significantly increased in patients compared to healthy controls, confirming the presence of systemic inflammation in CTS [49]. Here, we analyse a comprehensive set of blood inflammatory mediators at both mRNA and protein level 1) in the active stage of nerve injury (CTS pre-surgery versus healthy controls) and 2) in recovery (paired patient samples pre- and post-surgery). We also explore associations of inflammatory markers with patients’ symptoms and specifically neuropathic pain.

## Methods

### Participants

We used the data available from the prospective longitudinal Oxford CTS cohort [2]. Patients with clinically and electrodiagnostically confirmed CTS were recruited from surgical waiting lists at Oxford University Hospitals NHS Foundation Trust. Patients were excluded if electrodiagnostic testing revealed a nerve dysfunction other than CTS, if there was another medical condition affecting the upper limb/neck (e.g. hand osteoarthritis, cervical radiculopathy), if there was a history of significant trauma to the upper limb or neck, or if they were pregnant. Patients with potentially confounding conditions such as autoimmune or inflammatory disease (e.g., rheumatoid arthritis, multiple sclerosis), active infection (e.g., hepatitis), other systemic illnesses (e.g., diabetes, cancer), or those taking immunosuppressive medications were also excluded. Patients undergoing repeat CTS surgery were also excluded. Patients with CTS were assessed at baseline (before surgery) and six months following surgery, when functional and structural neural recovery as well as symptom improvement is apparent [2] and systemic cytokine levels are unlikely to be influenced by a potential inflammatory reaction related to the surgical intervention.

Twenty-one, age and gender matched, healthy controls were also included in the study. They did not have any systemic illness, including potentially confounding conditions mentioned above, did not experience pain in the hand in the past 3 months, and electrodiagnostic testing of the radial, ulnar and median nerve were within normal limits. Healthy participants were recruited within the university department, via public notice boards and media advertisements. All healthy controls attended one assessment. Ethical approval was given for the project (Riverside London ethics committee Ref 10/H0706/35) and all participants provided informed written consent prior to participating.

### Phenotypic data

A detailed description of the phenotypic data collected is available elsewhere [2]. For this study, we included age, sex, height, weight and body mass index as baseline variables. Symptom duration was recorded in months. Symptom severity was evaluated with the symptom subscale of the Boston carpal tunnel questionnaire [41] (0 = no symptoms, 5 = severe symptoms). Neuropathic pain severity was evaluated with the Neuropathic Pain Symptom Inventory (NPSI) [8], which includes numerical rating scales (0 = no pain to 10 = worst pain imaginable) for burning pain, deep pressure pain, paraesthesia, paroxysmal pain, evoked pain, as well as a composite score (0-100). The severity of pain over the past 24 hours was recorded on a visual analogue scale (VAS; 0 = no pain, 10 = worst pain imaginable). Surgical outcome was determined with the Global Rating Of Change Scale, which ranges from -7 (a very great deal worse) to +7 (a very great deal better) [32]. A patient was considered to have a successful recovery after surgery if they reported a GROC score of ≥ +5 (a good deal better) [35]. Standard electrodiagnostic testing (EDT) of the median, ulnar and radial nerve was performed with an ADVANCE™ system (Neurometrix, USA). Electrodiagnostic test severity was graded on the scale derived by Bland et al, 2000 [6] as follows: normal (grade 0), very mild (grade 1), mild (grade 2), moderate (grade 3), severe (grade 4), very severe (grade 5), extremely severe (grade 6). For a more detailed description of the electrodiagnostic testing see Schmid et al [60].

### Blood sampling and processing

Three millilitres of venous blood was sampled into RNA stabilising tubes (Tempus™ blood RNA tube, Fisher Scientific, UK) and stored at -20°C for batch processing. Blood serum was extracted from whole blood collected into a BD Vacutainer® SST™ tube for serum collection (BD, UK). The blood was left to clot before being centrifuged at 3000 rpm for 10 minutes at 4° C. The serum fraction was aliquoted and stored at -80°C for batch processing.

### Gene expression

RNA was extracted from blood following published protocols (Tempus™ Spin RNA Isolation Kit). Briefly, samples were defrosted and PBS added to each sample which was then vortexed and centrifuged. The RNA pellet was re-suspended and purified via column filtration. RNA was converted into cDNA using the EvoScript Universal cDNA Master kit (Roche, UK). Custom made TAQMAN array microfluidic cards (ThermoFisher, UK) were designed containing 44 markers implicated in inflammation and/or neuropathic pain as well as three housekeeping genes. TAQMAN array cards were used as they are highly sensitive [45] and use a pre-loaded assay format, with the remaining master mix and sample being added through specialised loading ports, which substantially reduces operator error. The gene list contained cytokines and chemokines, both anti- and pro-inflammatory and immune cell markers such as CD3D, CD16 and CD14 to detect the presence of T cells, neutrophils and monocytes respectively. The full list of genes can be found in Supplementary Table 1. The cards were run as per standard protocol. In brief, 60μl of patient cDNA was mixed with 60μl of TAQMAN Fast Advanced Master Mix to achieve a final volume of 120μl and cDNA concentration of 10ng/μl. Paired patient samples from before and after surgery were processed on the same card with each assay being run in singlet. The cards were run on a Quantstudio 7 Flex Real-Time PCR System (Applied Biosystems, USA). Cycle times (Ct) for each gene in each sample were recorded and used in future analyses. We included TRAP1 and DECR1 as housekeeping genes, as they are stably expressed in human blood [52,65]. 18S was also included as a mandatory housekeeping gene on the microfluidic card. The average expression of TRAP1, DECR1 and 18S were subtracted from the expression of the genes of interest to provide normalised expression values.

### Protein levels

U-PLEX plate custom biomarker multiplex assay kits (Meso Scale Diagnostics LLC, USA) were custom designed to detect 18 selected cytokines/chemokines related to the gene expression data with high sensitivity (Supplementary Table 1) [12,16]. Processing followed the standard manufacturer protocols. Each capture antibody was combined with a specific linker molecule and incubated at room temperature for 30 minutes. The linking reaction was inhibited with the addition of stop solution. Linked capture antibodies were pooled and 50μl was added to each well and incubated at 4°C overnight on a shaker. The next day, the capture antibody solution was removed and the plate was washed three times with PBS +0.05% Tween 20, followed by addition of 25μl assay buffer along with either 25μl serum or assay standards. The plates were incubated on a shaker at room temperature for 1 hour. Patient samples and standards were removed and the wells were washed 3x with wash buffer. Fifty μl of detection solution was added to each well before incubation for 1 hour at room temperature on a shaker. The plates were washed 3x with wash buffer and 150μl of read buffer was added before the plates were read according to the manufacturer’s instructions on a MESO QuickPlex SQ 120 plate reader (Meso Scale Diagnostics LLC, USA).

For detection of TGF-β, the same procedure was followed, but acidification was required as per standard protocol: samples were treated with 20μl 1M HCl per 100ml and incubated at room temperature for 10 minutes before neutralising by the addition of 14μl of 1.2M NaOH in 0.5M HEPES buffer per 100μl of sample.

As CCL5 (RANTES) was not available in the U-plex panel, the detection of CCL5 was performed with R-PLEX plates (Meso Scale Diagnostics LLC, USA). The standard manufacturer protocol was used. The procedure was the same as described above with the exception that the serum samples were diluted 1:50. No specific linker molecules were used, instead, streptavidin coated plates were used to bind biotinylated anti CCL5 capture antibodies.

To detect Human C Reactive Protein (CRP) in serum, we used a CRP Quantikine ELISA kit (R&D Systems, US) and followed the standard protocol. Briefly, 100μl of assay diluent was added to each well, followed by 50μl of either standard or sample (serum samples were diluted 100-fold in calibrator diluent). We ran the samples along with an eight-point standard curve in duplicate. The plates were sealed and incubated at room temperature for 2 hours. Sample or standards were then aspirated off and wells were washed 4x with 400μl wash buffer. 200 μl of human CRP conjugate (secondary antibody with horseradish peroxidase activity) was added to each well and incubated at room temperature for 2 hours. The plates were then washed 4x with 400μl buffer and 200μl of substrate solution was added. The plates incubated at room temperature for 30 minutes in the dark. Fifty μl of stop solution was added and the plates were read on a BMG FLUOstar Omega (BMG Labtech Ltd, UK) with the wavelength set to 450 nm.

All patient samples were run in duplicate and paired patient samples (before and after surgery) as well as standards were processed on the same plate. Standards were composed of known concentrations of each cytokine/chemokine being analysed and were used to calculate the protein concentrations in each patient sample. Quantification of the target inflammatory mediators was based on duplicates of an 8-point calibration curve which was calculated automatically using discovery workbench software for MSD plates or interpolated from the standard curve in prism for CRP.

### Statistical Analysis

This analysis is a secondary analysis of a published cohort [2] of exploratory character and did therefore not include an a-priori sample size calculation [29]. Genomic data were analysed with the statistics package R [70], using the software package limma [57] to determine differential gene expression. Batch correction was conducted on normalised data sets. The batch corrected data were input into an empirical Bayesian statistical model to shrink variance within the data. Data were then fitted into a mixed linear model to determine differential gene expression using patient as blocking factor. Differential expression was investigated 1) between patients before surgery and healthy controls (active stage of nerve injury) and 2) between patients before and after surgery (recovery). Results were corrected for false discovery rate (FDR) with Benjamini-Hochberg correction where an adjusted p value of <0.05 was considered significant.

Mann-Whitney U tests were used to compare normalised serum protein levels between healthy participants and patients with CTS before surgery (active stage of nerve injury). Wilcoxon signed rank tests were conducted to compare serum protein levels between patients with CTS before and after surgery (recovery). Results were corrected for false discovery rate using Benjamini-Hochberg correction where an adjusted p value of <0.05 was considered significant. Any protein remaining significant after FDR correction was included in further analyses.

To determine associations between gene/protein expression and clinical phenotype, Spearman’s rank correlation analyses were conducted. Non-normal and zero-inflated data dictated the usage of the non-parametric and not sensitive to outliers rank-order Spearman’s correlation to assess the strength of monotonic relationships. Both before and after surgery data were used for most analyses. The only exception was for duration of symptoms (where only pre-surgery data were used), and GROC scores (where only post-surgery data were used). To limit the number of potential correlations and therefore type 1 error, only the two genes with largest Log2 fold change (Log2FC) and that were significant after FDR correction in each analysis were used in further analyses to determine associations with phenotype. In some instances, either the mRNA expression value or the protein level was not able to be determined for a particular inflammatory mediator, in any given patient. As such, some data points in the correlation analysis were not available. Scatter plots between mRNA expression or protein levels and clinical phenotype scores were first inspected to determine a monotonic relationship. Spearman’s correlation was only conducted for monotonic data. Several of the clinical scores included zero values. These values represent genuine sampling points for instance reflecting complete symptom resolution and were thus retained. Spearman’s correlation can cope well with zero inflated data of up to 30% [30]. However, to fully determine the effect of zero values, we used a hurdle model consisting of a truncated Poisson model fitted to non-zero scores and a binomial model fitted to zero scores. We used the same approach to determine potential associations between pre and post-operative changes in inflammatory markers and clinical phenotypes comparing Log2 fold changes of gene/protein levels and clinical phenotype scores. As these investigations were exploratory in nature, used a small sample size, did not involve repetitive hypothesis testing, and because exploratory correlations were carried out for inter-dependent variables, FDR correction was not suitable in this instance.

The effects of age, gender and BMI on the mRNA and protein expression of inflammatory mediators were found to be very limited (Supplementary Tables 2 & 3) after fitting a mixed linear model or preforming a two-way ANOVA including covariates for age, gender and BMI on mRNA and protein expression data, respectively. As these covariates were uninformative, they were not included in the differential expression analysis or in correlations between inflammatory mediator expression and clinical phenotype data. Their inclusion would have added unnecessary complexity to the models, reducing power and diluting effects. In the case of post-versus pre-surgery comparison, the usage of a mixed linear model controls for individual differences in baseline expression and consequently for variance due to differences in individuals’ age, gender or BMI.

## Results

Baseline and clinical data of the 55 patients with CTS and 21 healthy controls can be found in Table 1. Patients and controls were comparable for age, gender, height, weight and BMI (Table 1). After surgery, 47 patients (85%) were classified as successfully recovered as they reported a GROC score of ≥ +5.

### Systemic inflammatory changes in the active stage of nerve injury

Table 2 contains the results of the gene expression analyses in the active stage of CTS compared to healthy controls. Differential expression analysis between healthy controls and patients with CTS pre-surgery revealed expression of a single gene: *PTGES2*: encoding a membrane bound enzyme which catalyses the conversion of Prostaglandin H2 to Prostaglandin E2, to be significantly decreased in patients compared to healthy controls (adjusted p=0.013).

**Figure 1:**
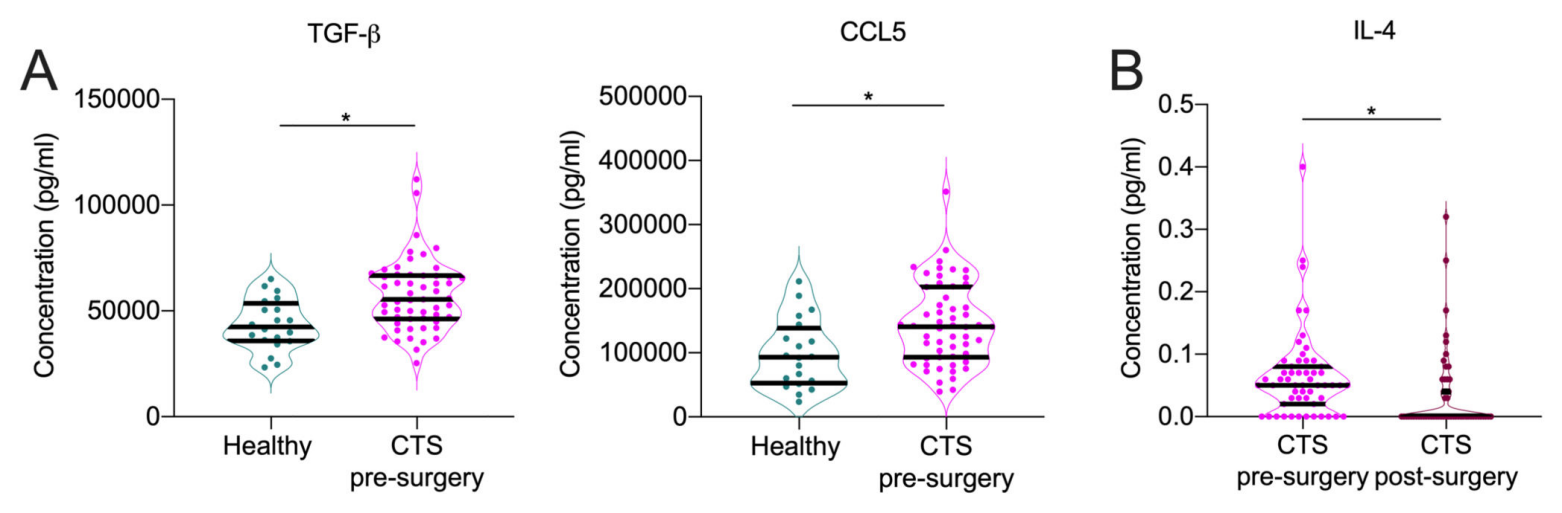
Significant changes in serum inflammatory protein levels. (A) Graphs show significantly increased serum protein levels for TGF-b and CCL5 in patients with CTS pre-surgery (pink) compared to healthy control (green). (B) Graph shows significantly downregulated IL-4 in patients with CTS after (red) compared to before surgery (pink). Data are shown as violin plots with median, quartiles and single data points. Significant dysregulation (FDR corrected Mann-Whitney U tests and Wilcoxon test respectively) is indicated with ^*^P<0.05.

The concentrations of inflammatory proteins in patients with CTS before surgery compared to healthy participants revealed two mediators that were significantly different. Both TGF-β (adjusted p=0.016) and CCL5 (adjusted p=0.047) were increased in patients with CTS pre-surgery compared to healthy controls (Figure 1, Table 3).

### Systemic inflammatory changes associated with resolution of the disease

Table 4 contains the paired gene expression analysis related to the resolution of CTS. A total of 12 genes were differentially expressed: *IL-9, CCL5, PDGFA, IL-1β, CXCL5, TGFB1, VEGFA, IL-4, TLR4, FCGR3B, IL-6* and *CD3D*. Of these, the two genes with the largest Log2FC were *IL-9* and *IL-6*, where *IL-9* mRNA was increased after surgery (Log2FC=-1.099) and *IL-6* mRNA was decreased after surgery (Log2FC=0.92). These 2 genes were included in further analyses.

Paired analysis of serum inflammatory protein levels in patients with CTS from before and after surgery identified IL-4 as being increased pre-surgery (adjusted p=0.002, Figure 1, Table 5) with no other marker being significantly different. The cytokine IL-9 which showed significantly different expression at the mRNA level, displayed a similar trend at protein level, although this was not significant after stringent FDR correction (adjusted p=0.09). IL-6 was not found to be significantly different at the protein level (adjusted p=0.24).

Supplementary Table 4 provides a summary of mRNA expression and protein levels in healthy controls and patients with CTS before and after surgery.

### Systemic inflammatory mediators correlate with clinical pain phenotypes

**Figure 2:**
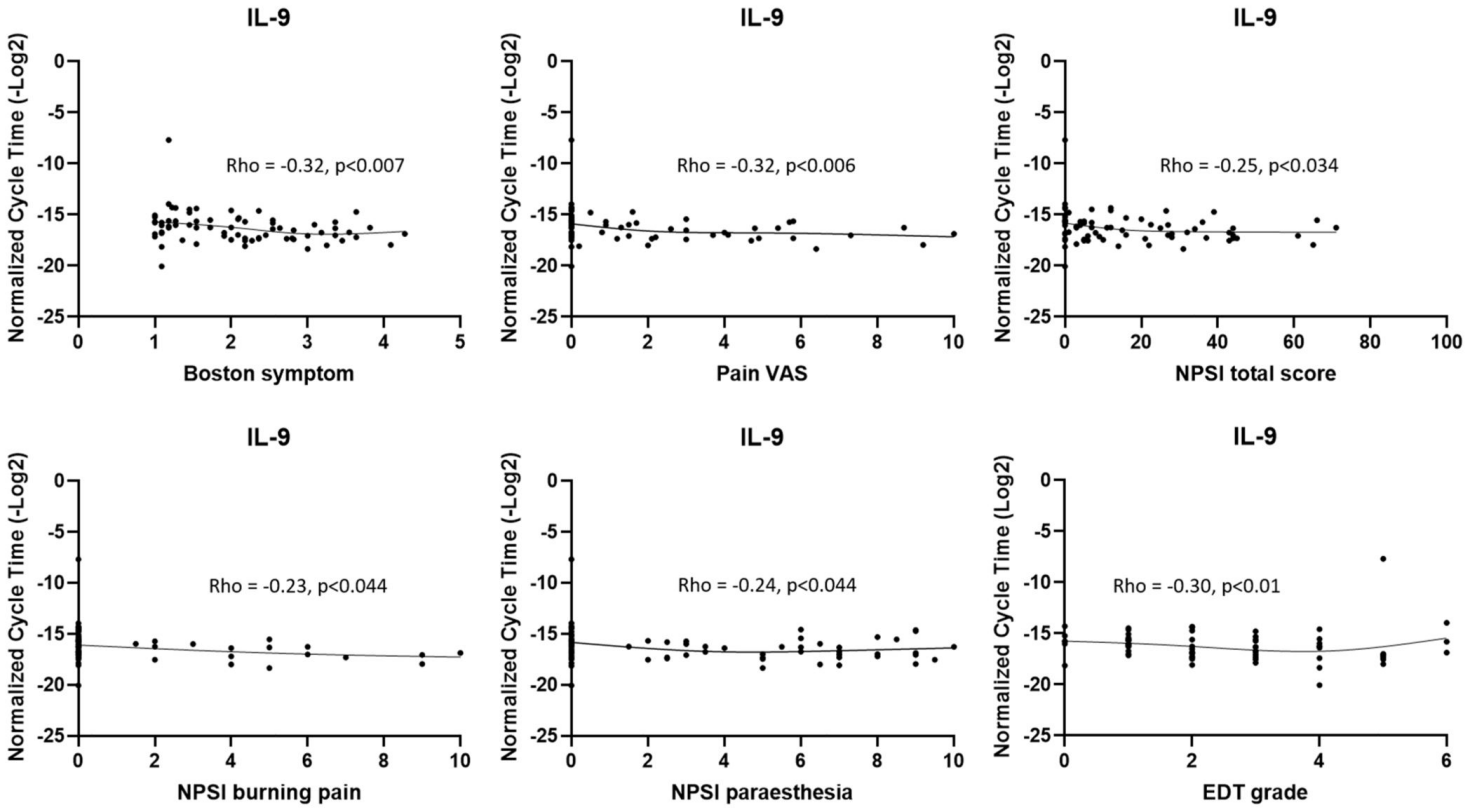
Significant correlations of inflammatory gene expression and clinical phenotypes. *IL-9* negatively correlated with a range of symptom scores (Boston symptom questionnaire, pain VAS, NPSI total score and sub-scores for burning pain and paraesthesia) as well as electrodiagnostic test severity (EDT grade). Pre- and post-surgery data were included in the analyses. Spearman’s rank correlation was used with a p<0.05 being considered significant. A smoothed spline has been added to highlight the trend of the data. VAS = Visual analogue scale, NPSI = Neuropathic pain symptom inventory

Results of the mRNA-clinical phenotype correlation analyses can be found in Figure 2. Correlation analyses revealed a significant correlation of *IL-9* mRNA expression with several pain scores. There was a negative correlation of *IL-9* mRNA with the Boston symptom score, VAS Pain and the NPSI composite as well as several sub-scores (burning pain and paraesthesia). *IL-9* mRNA expression also negatively correlated with EDT grade. For *IL-6* and *PGTES2* mRNA expression, no monotonic relationships were present with clinical phenotypes and so no correlation analyses were conducted for these mediators.

**Figure 3:**
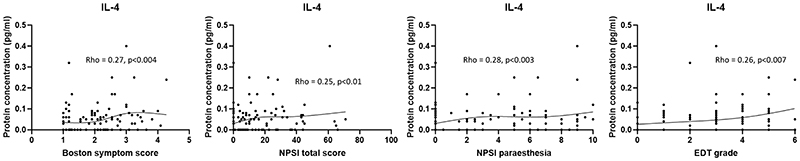
Significant correlations of protein levels and clinical phenotype. IL-4 protein levels positively correlated with the Boston symptom score as well as the NPSI total score and its subdomain paraesthesia. In addition, there was a positive correlation between IL-4 protein levels and electrodiagnostic test severity (EDT grade). Pre and post data were included in the analyses. Spearman’s rank correlation was used with a p<0.05 being considered significant. A smoothed spline has been added to highlight the trend of the data. VAS = Visual analogue scale, NPSI = Neuropathic pain symptom inventory

We next conducted correlation analyses between protein concentrations and patient phenotype scores. No monotonic relationships were found between protein level and patient phenotype scores for CCL5 or TGF-β and so analyses were not conducted for these mediators. IL-4 correlated positively with EDT grade and several pain scores including the Boston symptom questionnaire and the NPSI total score (Figure 3). Interestingly, when correlations were done using IL-9 protein concentrations it was again found to negatively correlate with the total NPSI score as well as two NPSI sub-scores (paraesthesia, paroxysmal pain) (Supplementary Figure 1).

To determine the effect of zero-inflation in these correlations, a hurdle modelling approach consisting of a truncated Poisson model for non-zero scores and a binomial model for zero scores was fit to the data (Supplementary Table 5). In general, this modelling was in good agreement with the Spearman’s correlation analysis, especially for *IL-9* mRNA expression, where the odds ratio showed that higher *IL-9* mRNA expression was associated with patients scoring zero for symptom severity. However, in the majority of these cases the correlation for the zero-inflated model did not reach significance.

To determine whether changes in mRNA expression or protein level were associated with changes in clinical symptoms, we analysed the Log2 fold change of *IL-6, IL-9* and *PTGES2* mRNA expression and CCL5, TGF-β, IL-4 and IL-9 protein levels against the Log2 fold change of clinical symptoms. However, no monotonic relationships were present for any of these mediators with clinical scores and thus no correlation analyses were conducted with Log2 fold change data.

## Discussion

Using CTS as a model system, we have explored changes in systemic blood inflammatory markers associated with the presence of nerve injury, neuropathic pain and recovery. In the active stage of CTS, *PTGES2* mRNA expression was significantly lower and TGF-β and CCL5 protein levels higher in patients compared to healthy controls, however large variation was observed in protein levels within groups. During recovery, 12 genes were significantly differentially expressed, among which *IL-9* (increased post-surgery) and *IL-6* (decreased post-surgery) showed the most pronounced changes. At protein level, IL-4 was significantly increased pre-surgery. Intriguingly, correlation analyses identified IL-9 to be negatively correlated with several pain scores at both the mRNA and protein level, while protein concentrations of IL-4 positively correlated with patients’ pain scores. Our findings highlight the potential role of systemic immune dysregulation in focal nerve injury and neuropathic pain.

### Systemic inflammatory changes associated with recovery

CTS provided a unique opportunity to study changes in inflammatory markers associated with resolution of nerve injury and neuropathic pain. The most striking finding of the pre- to post-surgery comparison was that of IL-9, which showed a significant increase in mRNA expression post-surgery, a similar trend at protein level and consistent negative associations with a range of pain severity scores. The post-operative phase of CTS is reflective of a state of recovery, where symptoms have largely resolved and the affected nerve is in the process of repair [2]. The post-operative increase in IL-9 may therefore highlight a role for this cytokine in symptom resolution and nerve repair/regeneration. IL-9 is a relatively understudied cytokine with limited literature describing a pleiotropic function with both pro and antiinflammatory capacity [27,33,46,66]. Studies in patients with inflammatory bowel disease report that increased IL-9 serum protein concentration correlated with a less favourable prognosis and increased disease severity [19,71]. In contrast, but in line with our findings, other studies point towards a pro-resolution effect of IL-9. Compelling preclinical data implicates IL-9 in the activation and activity of Treg cells [21,46,56]. A pro-resolution role of IL-9 has also been confirmed in patients with rheumatoid arthritis in clinical remission [56] and in patients with lumbar radicular pain, where IL-9 was higher in patients with mild compared to severe disc herniations [31]. These recent discoveries, together with our findings, suggest that IL-9 may be an interesting candidate with a pro-resolution role in the context of neuropathic pain. Further work may seek to determine whether IL-9 has therapeutic utility in neuropathic pain.

In contrast to IL-9, IL-6 gene but not protein expression was higher before compared to after CTS surgery. IL-6 is a well-studied pro-inflammatory cytokine with a clear link to pain, including neuropathic pain [84]. Whereas the literature on IL-6 levels in serum of patients with CTS is conflicting [26,36,49,69], its contribution to other entrapment neuropathies such as lumbar radicular pain and its severity is well established [37,54,59,61,77]. The therapeutic utility of IL-6 in the treatment of neuropathic pain has already started to be explored. For instance, tocilizumab, a humanised antibody which binds the IL-6 receptor, has shown promising short-term effects in preliminary clinical trials in patients with sciatica [51,58].

IL-4 protein levels were also increased before compared to after surgery. Preclinical literature suggests that IL-4 is a key mediator in reducing neuropathic pain behaviours in models of peripheral nerve injury [7,75]. This contrasts with our findings of IL-4 downregulation in the resolution phase and its positive correlation with several pain scores. Of note, IL-4 was changed at both mRNA and protein levels albeit in opposing directions. This discrepancy in mRNA expression and protein levels is commonly observed [18,48] and is thought to be driven in large part by protein half-life [3,81]. Cytokines have typically very short half-lives [10,79], which could explain the observed discrepancy. However, the serum levels were low (<0.5 pg/ml) and the clinical relevance of IL-4 remains therefore unclear.

### Systemic inflammatory changes in the active stage of nerve injury

A limited number of systemic inflammatory mediators were altered in patients with CTS compared to healthy controls. This is most likely attributed to the vast variation in cytokine expression in humans [42], which requires either large effects or large samples to detect subtle changes. Indeed, CTS represents a focal and more subtle nerve injury compared to more severe neuropathic conditions such as phantom limb pain or systemic inflammatory neuropathies where pro-inflammatory cytokines are often dysregulated [13,53]. Nevertheless, we observed higher CCL5 and TGF-β at protein level and lower *PTGES2* mRNA levels in patients with CTS. In line with our findings, CCL5 has previously been identified as being increased in the serum of patients with CTS compared to healthy controls [49]. We did not detect any significant correlations for CCL5 with any pain phenotypes. CCL5 has however previously been implicated in the generation of neuropathic pain in experiments treating mice with the CCL5 antagonist met-RANTES [43] or by using CCL5 knockout mice [44]. Interestingly however, a previous study has found that serum CCL5 levels in CTS negatively correlated with neuropathic pain severity [49]. Given our replication of changes to CCL5 in CTS as a model system of neuropathic pain and the growing preclinical literature, the role of CCL5 in the context of nerve injury and neuropathic pain deserves more attention. TGF-β protein levels were also higher in patients with CTS compared to healthy controls. TGF-β has previously been implicated in the pathogenesis of CTS, but mainly with regards to fibrotic changes [15,23,28,62]. The here identified higher TGF-β levels may therefore be acting more as a driver for fibrotic change in CTS, rather than protecting against neuropathic pain. Of note, we have identified *TGFB3* as a causal gene in a genome wide association study of CTS [80], further corroborating the importance of this pathway.

Prostaglandin E2, has a well-established association with acute and chronic neuropathic pain in preclinical models [47]. In contrast to our findings of downregulated *PTGES2* mRNA expression in patients with CTS compared to controls, serum protein levels have been reported to be unchanged [26]. Nevertheless, analysis of tenosynovial tissue indicated that Prostaglandin E2 is increased in patients with CTS compared to healthy controls [5,26,72]. The apparent discrepancy between our findings and that of the literature may be due to differences in the biological samples used and the method of analysis. We analysed mRNA expression instead of protein levels, which often do not correlate [18,48].

### Many differentially expressed cytokines are involved in naïve CD4+ T-cell differentiation

An interesting feature of several of the dysregulated inflammatory mediators identified here (TGF-β, IL-4 and IL-6) is that they are known to be involved in naïve CD4+ helper T-cell differentiation [4,11,14,24,25,39,68,76,83]. They may therefore be working in combination to orchestrate specific sub-populations of CD4+ T-cells. Indeed, flow cytometric analysis of peripheral blood in patients with CTS identified increased CD4+ T-cell effector memory and central memory populations in patients with CTS compared to healthy controls [49]. These findings further fuel the increasing interest in T-cells in the context of neuropathic pain [38].

### Limitations

A limitation of this study is that the cellular sources of the cytokines and the target cells upon which the cytokines act remain unknown. This knowledge would prove useful in fully defining the role of inflammation in neuropathic pain and its recovery. Another issue with measuring cytokine expression systemically in the blood is that it may not accurately reflect the local environment at the lesion site and may miss markers that do not circulate at high levels in the blood. Nevertheless, our findings demonstrate that peripheral blood analysis can be informative in the context of neuropathic pain if lesioned tissues are not available. The protein analyses in particular showed high variation within groups, which may reflect differences in disease presentation as well as inherent differences in baseline expression of these mediators. The lower number of healthy participants may have contributed in part to this variation. Caution should thus be taken when interpreting the role of these mediators. However, as this study did not intend to identify biomarkers of CTS, the identification of specific biological pathways and mediators associated with the active stage of the disease and its recovery may still prove insightful. Finally, even though the list of inflammatory mediators investigated here is more comprehensive than in previous studies, it is not exhaustive and may have missed relevant markers. Our initial data will be an important resource to guide future validation studies.

## Conclusions

Investigating the systemic expression of inflammatory mediators in patients with carpal tunnel syndrome revealed a role both during the active stage as well as resolution of nerve injury and neuropathic pain. Intriguingly, IL-9 was upregulated during recovery and consistently negatively correlated with symptom scores, suggesting a pro-resolution role in the context of nerve injury and neuropathic pain. *PTGES2* mRNA as well as TGF-β and CCL5 protein levels were associated with the active stage of nerve injury, and *IL-6* and *IL-4* mRNA and protein levels were upregulated pre- compared to post-surgery respectively. Our findings implicate specific cytokines to play a role in neuropathic pain associated with focal nerve injury and its recovery.

## Supplementary Material

Supplementary files

## Figures and Tables

**Table 1:** Baseline and clinical data Data are presented as median with interquartile range [square brackets] unless indicated otherwise

	Healthy	CTS pre	CTS post
Number of Participants	21	55	55
Age (years)	63 [21]	64 [16]	
Female gender, n (%)	14 (66.7 %)	37 (67.3 %)	
Mean height (SD) [cm]	169.12 (9.34)	168.19 (8.80)	
Weight (kg)	70.4 [15.75]	68.7 [16.8]	
BMI (kg/m^2^)	26 [5]	24 [5]	
Duration of symptoms		36 [42]	
(months)			
EDT grade		3 [2]	2 [2]
Normal, n (%)		0 (0)	7 (13)
Very mild, n (%)		4 (7)	18 (33)
Mild, n (%)		7 (13)	9 (17)
Moderate, n (%)		18 (33)	11 (20)
Severe, n (%)		11 (20)	7 (13)
Very severe, n (%)		13 (24)	2 (4)
Extremely severe, n (%)		2 (4)	1 (2)
Boston symptom score		2.55 [1.12]	1.27 [0.45]
Boston function score		2.13 [1.19]	1.25 [0.63]
VAS pain		1.7 [4.05]	0 [0]
NPSI total score		21 [25]	1 [7.5]
burning pain		0 [4]	0 [0]
deep pain		1.5 [2.75]	0 [0]
evoked pain		0.33 [2.67]	0 [1]
paraesthesia		6 [4.75]	0 [0]
paroxysmal pain		0 [3]	0 [0]
GROC Score			7 [1]

SD = standard deviation, IQR = interquartile range, EDT = electrodiagnostic testing, VAS = visual analogue scale, NPSI = Neuropathic pain symptom inventory, GROC = global rating of change.

**Table 2:** Gene expression changes in the active stage of CTS Changes in gene expression in patients with CTS (pre-surgery) compared to healthy controls. Genes are ranked in descending order based on adjusted p values. Significant dysregulation (adjusted p<0.05) is indicated with grey shading.

Gene	LogFC	AveExpr	P Value	Adj.p.Val
*PTGES2*	0.72	7.35	0.000	0.013
*FCGR3B*	-0.99	6.71	0.002	0.052
*IL4*	-0.64	11.08	0.004	0.055
*CXCL5*	-0.88	6.21	0.005	0.055
*IL23A*	0.36	9.26	0.011	0.076
*CCL5*	-0.42	1.06	0.013	0.076
*CXCL8*	-0.76	6.59	0.012	0.076
*IL12B*	-0.94	16.60	0.008	0.076
*IL1B*	-0.43	5.96	0.022	0.095
*TLR4*	-0.43	5.15	0.019	0.095
*TGFB1*	-0.22	2.59	0.058	0.224
*PDGFA*	-0.39	9.65	0.061	0.224
*CXCL10*	0.50	10.69	0.071	0.245
*IL9*	-0.61	16.86	0.079	0.254
*IFNG*	-0.33	9.73	0.114	0.303
*MMP9*	-0.36	4.82	0.103	0.303
*IL6*	-0.97	11.83	0.116	0.303
*CX3CL1*	-2.56	15.64	0.123	0.303
*IL22*	-4.55	14.22	0.126	0.303
*CRP*	2.04	15.74	0.156	0.357
*CCL2*	0.40	13.04	0.181	0.377
*CCL21*	5.59	15.30	0.212	0.424
*CHI3L1*	-0.41	6.04	0.240	0.462
*NOS2*	-0.69	15.83	0.253	0.467
*IL10*	0.28	13.87	0.278	0.480
*CD80*	-0.22	11.57	0.280	0.480
*IL7*	-0.16	9.58	0.423	0.700
*TNF*	-0.20	6.43	0.443	0.709
*CD14*	0.11	8.27	0.471	0.729
*VEGFA*	0.09	9.72	0.512	0.744
*CCL4*	-0.15	6.95	0.500	0.744
*CXCL11*	0.13	11.72	0.528	0.746
*CD3D*	0.08	4.39	0.546	0.746
*IL13*	-0.18	16.15	0.606	0.746
*IL17A*	-1.15	15.82	0.605	0.746
*CCL11*	-8.98	7.21	0.594	0.746
*TAC1*	4.44	-1.08	0.640	0.749
*NGF*	3.83	8.19	0.674	0.770
*IL18*	-0.15	6.83	0.716	0.799
*IL1RN*	1.05	11.79	0.808	0.881
*IL2*	-0.04	13.30	0.849	0.891
*CXCL9*	-0.05	11.30	0.854	0.891
*CSF3*	0.39	14.03	0.913	0.932
*IL5*	0.09	13.66	0.974	0.974

LogFC = Log2 Fold Change, AveExpr = Average Expression (Average normalised Ct value across all samples), Adj.p.Val = Adjusted P value.

**Table 3:** Serum level changes in the active stage of CTS Differences in serum inflammatory marker levels in patients with CTS (pre-surgery) compared to healthy controls. Inflammatory mediators are ranked in descending order based on adjusted p values. Significant dysregulation (p<0.05) is indicated with grey shading. Values are given in pg/ml apart from CRP which is provided in mg/l

	Healthy		CTS (pre surgery)			
Inflammatory mediator	Median	IQR	Median	IQR	P Value	Adj.p.Value
TGF-β	42447.69	15597.64	55410.50	20339.69	0.0008	0.016
CCL5	93212.10	72600.04	140804.24	100719.45	0.005	0.047
IL-4	0.01	0.05	0.05	0.06	0.040	0.267
CXCL10	190.02	114.23	233.83	158.86	0.092	0.460
CCL2	263.16	82.47	288.87	122.57	0.127	0.488
VEGF	74.75	50.40	94.88	81.73	0.147	0.488
IL-10	0.20	0.16	0.17	0.13	0.198	0.523
CXCL8	7.90	4.14	9.03	5.13	0.209	0.523
IL-6	0.80	0.31	0.66	0.62	0.370	0.812
IL-9	0.31	0.23	0.23	0.27	0.406	0.812
IL-2	0.00	0.00	0.00	0.00	0.491	0.857
IFN-γ	6.69	7.49	6.65	5.67	0.546	0.857
IL-17	0.00	0.00	0.00	0.00	0.693	0.857
IL-1β	0.08	0.07	0.09	0.07	0.727	0.857
CRP	1.27	2.70	1.49	1.54	0.728	0.857
GM-CSF	0.00	0.01	0.00	0.04	0.743	0.857
TNF-α	0.56	0.29	0.50	0.35	0.751	0.857
CXCL5	1565.00	1467.27	1533.24	1068.58	0.771	0.857
IL-12	0.11	0.30	0.00	0.30	0.872	0.899
Fractalkine	6061.66	1333.26	6149.32	2099.72	0.899	0.899

IQR = Interquartile range, Adj.p.Value = Adjusted P value.

**Table 4:** Gene expression changes associated with resolution of CTS Changes in gene expression in patients with CTS before compared to after surgery. Genes are ranked in descending order based on adjusted p values. Significant change (adjusted p<0.05) is indicated with grey shading where genes are ranked by Log2 fold change.

Gene	LogFC	AveExpr	P Value	Adj.p.Val
*IL-9*	-1.10	16.26	0.0003	0.014
*IL-6*	0.92	12.02	0.008	0.034
*CXCL5*	-0.55	5.69	0.002	0.027
*FCGR3B*	-0.45	6.22	0.006	0.031
*IL-1β*	-0.33	5.68	0.002	0.027
*TLR4*	-0.31	4.88	0.006	0.031
*IL-4*	-0.31	10.75	0.006	0.031
*PDGFA*	-0.27	9.41	0.003	0.027
*CCL5*	-0.24	0.83	0.003	0.027
*VEGFA*	-0.20	9.64	0.007	0.031
*CD3D*	0.17	4.50	0.010	0.039
*TGFβl*	-0.16	2.46	0.005	0.031
*IL10*	-0.29	13.80	0.026	0.095
*IL13*	-0.38	15.89	0.029	0.100
*MMP9*	-0.25	4.59	0.031	0.101
*CHI3L1*	-0.28	5.79	0.058	0.175
*CXCL10*	-0.24	10.71	0.064	0.181
*NOS2*	-0.86	15.23	0.109	0.275
*CXCL8*	-0.18	6.29	0.149	0.357
*CX3CL1*	1.48	15.62	0.181	0.414
*IL18*	-0.32	6.63	0.227	0.495
*CSF3*	2.02	15.55	0.260	0.543
*IL22*	2.21	13.88	0.306	0.612
*CCL11*	15.43	9.36	0.394	0.727
*TNF*	0.10	6.42	0.449	0.799
*TAC1*	6.55	0.49	0.545	0.837
*IL2*	-0.07	13.26	0.540	0.837
*CCL2*	-0.08	13.11	0.558	0.837
*IL12B*	-0.18	16.10	0.587	0.854
*NGF*	1.05	10.73	0.773	0.863
*IL1RN*	0.97	12.98	0.694	0.863
*CRP*	0.13	16.46	0.716	0.863
*CXCL11*	0.03	11.77	0.769	0.863
*CD14*	0.03	8.31	0.729	0.863
*IL7*	-0.03	9.52	0.739	0.863
*CD80*	-0.04	11.49	0.717	0.863
*CXCL9*	-0.05	11.26	0.708	0.863
*PTGES2*	-0.05	7.53	0.638	0.863
*IL17A*	-0.72	14.82	0.642	0.863
*IL5*	0.19	13.78	0.903	0.975
*CCL21*	0.09	16.52	0.955	0.975
*CCL4*	0.01	6.91	0.947	0.975
*IFNG*	-0.01	9.63	0.921	0.975
*IL23A*	0.00	9.36	0.978	0.978

LogFC = Log2 Fold Change, AveExpr = Average Expression (Average normalised Ct value across all samples), Adj.p.Val = Adjusted P value.

**Table 5:** Serum level changes associated with resolution of CTS Differences in serum levels in patients with CTS before compared to after surgery. Inflammatory mediators are ranked in descending order based on adjusted p values. Significant dysregulation (p<0.05) is indicated with grey shading. Values are given in pg/ml apart from CRP which is provided in mg/l

	Pre-Surgery		Post-Surgery			
Inflammatory mediator	Median	IQR	Median	IQR	P value	Adj.p.Value
IL-4	0.05	0.06	0.00	0.04	0.0001	0.002
Fractalkine	6149.32	2099.72	6246.77	1809.57	0.008	0.078
IL-9	0.23	0.27	0.39	0.20	0.014	0.091
IL-12	0.00	0.30	0.00	0.14	0.034	0.171
IL-6	0.66	0.62	0.70	0.86	0.065	0.242
IL-1β	0.09	0.07	0.06	0.08	0.073	0.242
GM-CSF	0.00	0.04	0.00	0.09	0.129	0.368
IL-10	0.17	0.13	0.17	0.12	0.250	0.626
CCL2	288.87	122.57	299.20	154.29	0.482	0.817
TNF-α	0.50	0.35	0.53	0.33	0.493	0.817
IL-2	0.00	0.00	0.00	0.00	0.500	0.817
IL-17	0.00	0.00	0.00	0.12	0.566	0.817
CRP	1.49	1.54	1.77	1.96	0.581	0.817
VEGF	94.88	81.73	103.40	78.38	0.592	0.817
CXCL5	1533.24	1068.58	1438.87	991.59	0.644	0.817
CCL5	140804.24	100719.45	134049.52	107239.00	0.656	0.817
CXCL8	9.03	5.13	10.68	4.08	0.694	0.817
TGF-β	55410.50	20339.69	54827.34	20182.99	0.762	0.846
CXCL10	233.83	158.86	231.69	192.15	0.918	0.952
IFN-γ	6.65	5.67	6.21	4.03	0.952	0.952

IQR = Interquartile range, Adj.p.Value = Adjusted P value.
