# Supplementary material for: Systemic inflammatory markers in neuropathic pain, nerve injury and recovery
Source: Pain. Author manuscript; Available in PMC 2022 Mar 1. (PMC7612369; doi:10.1097/j.pain.0000000000002386)

Supplementary Table 1: List of inflammatory mediators examined at gene and protein level

Gene	Protein	Gene	Protein
IL-1β	IL-1β	*CXCL5*	CXCL5
IL-1RN/IL-1RA		*CXCL8*	CXCL8
IL-2	IL-2	*CXCL9*	
IL-4	IL-4	*CXCL10*	CXCL10
IL-5		*CXCL11*	
IL-6	IL-6	*CX3CL1/Fractalkine*	CX3CL1
IL-7		*CSF3/G-CSF*	GM-CSF
IL-9	IL-9	*VEGF-a*	VEGF
IL-10	IL-10	*NGF*	
IL-12b	IL-12	*TGF-β1*	TGF-β
IL-13		*PDGFA*	
IL-17a	IL-17A-F	*CRP*	CRP
IL-18		*TAC1/Substance P*	
IL-22		*PTGES2*	
IL-23		*MMP9*	
IFN-γ	IFN-γ	*CD3D*	
TNF-α	TNF-α	*CD14*	
CCL2	CCL2	*FCGR3B/CD16*	
CCL4		*CD80*	
CCL5	CCL5	*CHI3L1*	
CCL11		*TLR4*	
CCL21		*NOS2*	

Supplementary Table 2: Age, gender and BMI contributions to blood inflammatory mediator expression at mRNA level. Using a mixed linear model, differential gene expression analysis was conducted based on age, gender and BMI. Significant differences in gene expression have been shaded in grey, adjusted p<0.05. Genes are ranked by their adjusted p value.

Gene	Phenotype	LogFC	AveExpr	P value	Adj.p.val
IL-18	Age	0.82	6.63	0.030	0.854
IL-4		0.34	10.75	0.042	0.854
IL-2		0.31	13.26	0.063	0.854
CXCL8		0.30	6.29	0.096	0.854
CD80		0.25	11.49	0.109	0.854
IL-13		0.33	15.89	0.153	0.854
CXCL11		-0.21	11.77	0.171	0.854
IL23a		0.15	9.36	0.190	0.854
PDGFA		0.19	9.41	0.196	0.854
CD14		-0.15	8.31	0.198	0.854
IFNγ		-0.21	9.63	0.200	0.854
CD3D		0.13	4.50	0.222	0.854
IL-6		0.48	12.02	0.294	0.917
CSF3		2.03	15.55	0.323	0.917
IL-7		-0.15	9.52	0.329	0.917
TLR4		-0.15	4.88	0.344	0.917
MMP9		-0.16	4.59	0.356	0.917
IL-5		-1.77	13.78	0.363	0.917
CXCL9		-0.16	11.26	0.384	0.920
PTGES2		0.12	7.53	0.437	0.920
TNF		0.13	6.42	0.477	0.920
TGFB1		0.05	2.46	0.500	0.920
CCL4		-0.10	6.91	0.519	0.920
IL1RN		1.77	12.98	0.522	0.920
IL-1β		0.10	5.68	0.529	0.920
NGF		-2.58	10.73	0.546	0.920
FCGR3B		-0.13	6.22	0.597	0.920
NOS2		-0.38	15.23	0.605	0.920
CCL11		-9.95	9.36	0.606	0.920
TAC1		5.09	0.49	0.611	0.920
CXCL5		0.13	5.69	0.613	0.920
IL-10		0.08	13.80	0.668	0.943
IL-17a		0.76	14.82	0.717	0.964
IL-22		0.83	13.88	0.739	0.964
CXCL10		-0.05	10.71	0.777	0.964
CCL5		0.03	0.83	0.822	0.964
CHI3L1		-0.05	5.79	0.825	0.964
IL-12b		0.09	16.10	0.841	0.964

VEGFA		0.02	9.64	0.866	0.964
CCL2		-0.03	13.11	0.884	0.964
CX3CL1		0.10	15.62	0.942	0.978
CCL21		0.10	16.52	0.962	0.978
IL-9		0.02	16.26	0.962	0.978
CRP		0.01	16.46	0.978	0.978
CD3D	Gender	-0.38	4.50	0.000	0.013
IL-23a		-0.27	9.36	0.015	0.266
CXCL5		-0.57	5.69	0.032	0.266
CXCL8		-0.38	6.29	0.036	0.266
IL-1β		-0.32	5.68	0.040	0.266
IL-7		0.31	9.52	0.044	0.266
CCL5		-0.25	0.83	0.046	0.266
IL-13		-0.46	15.89	0.050	0.266
PDGFA		-0.29	9.41	0.051	0.266
IL-6		0.76	12.02	0.103	0.413
CCL2		-0.34	13.11	0.105	0.413
IL-17a		3.23	14.82	0.149	0.507
CD80		-0.22	11.49	0.168	0.528
CCL21		2.04	16.52	0.269	0.744
IL-22		2.64	13.88	0.292	0.762
PTGES2		-0.15	7.53	0.318	0.784
CXCL9		0.18	11.26	0.334	0.784
TGFβ1		-0.07	2.46	0.412	0.923
CCL4		-0.11	6.91	0.472	0.982
IL-5		-1.37	13.78	0.498	0.982
TAC1		6.67	0.49	0.501	0.982
CD14		0.08	8.31	0.530	0.996
CXCL10		-0.11	10.71	0.574	0.998
TNF		0.10	6.42	0.603	0.998
IL-9		-0.16	16.26	0.672	0.998
NGF		1.61	10.73	0.700	0.998
IL-2		-0.07	13.26	0.703	0.998
CRP		-0.16	16.46	0.742	0.998
IFNγ		-0.05	9.63	0.749	0.998
IL-4		-0.05	10.75	0.787	0.998
IL-18		-0.10	6.63	0.798	0.998
CHI3L1		0.05	5.79	0.839	0.998
MMP9		-0.03	4.59	0.847	0.998
NOS2		0.13	15.23	0.862	0.998
CSF3		-0.42	15.55	0.862	0.998
VEGFA		0.02	9.64	0.864	0.998
IL-12b		0.08	16.10	0.877	0.998
CX3CL1		-0.16	15.62	0.908	0.998
CXCL11		0.02	11.77	0.915	0.998
TLR4		-0.01	4.88	0.938	0.998
IL-10		-0.01	13.80	0.978	0.998
FCGR3B		0.00	6.22	0.991	0.998

IL1RN		0.01	12.98	0.998	0.998
CCL11		NA	9.36	NA	NA
CCL11	BMI	-23.56	4.22	0.021	0.638
CCL21		-5.43	16.05	0.054	0.638
IL-22		-7.40	12.75	0.058	0.638
TAC1		-18.95	0.58	0.071	0.638
IL-2		-0.38	13.29	0.080	0.638
NOS2		-0.88	15.64	0.212	0.915
CXCL5		-0.33	5.97	0.230	0.915
IL-12b		0.51	16.32	0.233	0.915
IL-5		-3.20	13.68	0.244	0.915
IL-9		0.35	16.74	0.256	0.915
PTGES2		0.23	7.55	0.295	0.915
CCL2		0.31	13.15	0.299	0.915
PDGFA		-0.20	9.54	0.307	0.915
IL-13		-0.34	16.10	0.331	0.915
CCL4		0.22	6.91	0.368	0.915
CCL5		0.16	0.95	0.394	0.915
TLR4		0.15	5.04	0.406	0.915
IFNγ		0.18	9.64	0.426	0.915
IL1RN		-3.78	12.10	0.433	0.915
CXCL11		0.15	11.76	0.454	0.915
TGFB1		-0.08	2.53	0.490	0.915
IL-4		-0.14	10.90	0.544	0.915
IL-7		0.13	9.53	0.555	0.915
IL-1β		-0.11	5.85	0.578	0.915
CSF3		2.64	14.20	0.582	0.915
CD3D		0.08	4.41	0.612	0.915
CD14		-0.08	8.30	0.635	0.915
IL-18		0.23	6.79	0.637	0.915
CXCL8		-0.11	6.38	0.649	0.915
CHI3L1		0.14	5.93	0.664	0.915
FCGR3B		0.14	6.44	0.668	0.915
IL-17a		-1.01	15.53	0.698	0.915
MMP9		-0.08	4.72	0.714	0.915
CXCL1		0.79	14.77	0.742	0.915
CXCL10		0.07	10.83	0.787	0.915
CXCL9		-0.05	11.28	0.845	0.915
CRP		-0.13	16.35	0.856	0.915
CD80		0.04	11.51	0.858	0.915
IL-23a		-0.03	9.36	0.859	0.915
TNF		0.05	6.37	0.860	0.915
VEGFA		0.02	9.74	0.869	0.915
NGF		-1.16	8.83	0.877	0.915
IL-10		0.02	13.95	0.936	0.956
IL-6		0.03	11.56	0.972	0.972

LogFC = Log2 Fold Change, AveExpr = Average Expression (Average normalised Ct value across all samples), Adj.p.Val = Adjusted P value.

Supplementary Table 3: Age, gender and BMI contributions to serum inflammatory protein levels. Using two-way ANOVAs, the contribution of age, gender and BMI on the levels of inflammatory mediators in blood serum was investigated. F-statistics and P-values are shown, significant interactions are shaded in grey, P<0.05.

Protein	Age		Gender		BMI		Age:Gender:BMI	
	F	P	F	P	F	P	F	P
IL-1β	0.35	0.553	0.00	0.995	0.24	0.628	0.15	0.705
IL-2	0.01	0.906	0.36	0.547	0.01	0.916	0.18	0.670
IL-4	0.09	0.766	0.66	0.418	0.81	0.370	0.86	0.357
IL-6	0.26	0.615	0.18	0.673	0.18	0.673	0.23	0.634
IL-9	2.78	0.098	1.21	0.274	2.22	0.139	2.28	0.134
IL-10	0.62	0.431	0.28	0.595	0.04	0.848	0.08	0.778
IL-12	0.01	0.945	1.60	0.209	1.19	0.279	1.39	0.241
IL-17	5.55	0.020	7.49	0.007	5.07	0.027	8.21	0.005
IFN-γ	0.22	0.641	0.10	0.759	0.13	0.718	0.02	0.903
TNF-α	0.04	0.849	0.40	0.530	0.34	0.560	0.31	0.578
CCL2	3.66	0.058	0.06	0.806	0.36	0.548	0.20	0.659
CCL5	0.19	0.661	0.38	0.537	1.04	0.310	0.40	0.527
CXCL5	0.00	0.997	0.08	0.784	0.25	0.616	0.10	0.753
CXCL8	0.00	0.997	0.72	0.400	0.24	0.628	1.14	0.289
CXCL10	2.80	0.097	0.52	0.474	1.18	0.281	1.20	0.276
Fractalkine	0.27	0.602	0.86	0.355	0.05	0.830	0.20	0.657
GMCSF	1.65	0.201	2.47	0.119	1.67	0.199	2.29	0.133
VEGF	0.17	0.680	0.05	0.822	0.01	0.925	0.13	0.715
TGF-β	0.94	0.334	1.00	0.320	0.12	0.734	0.35	0.556
CRP	2.98	0.087	0.36	0.550	0.36	0.550	0.57	0.452

BMI = Body mass index

Supplementary Table 4: Summary of mRNA expression and protein levels in patients with CTS and healthy controls.

Average normalised cycle times (Ct) and (standard deviation) are shown for the mRNA expression of inflammatory mediators in healthy controls and patients with CTS pre and post-surgery. The corresponding median protein levels (pg/ml) and [IQR] are shown for healthy participants and patients with CTS pre and post-surgery. Protein levels of CRP are shown in mg/ml.

Gene (Ct)	Healthy	CTS pre-surgery	CTS post-surgery	Protein (pg/ml)	Healthy	CTS pre-surgery	CTS post-surgery
IL-1β	6.27 9 (0.66)	5.85 (0.72)	5.51 (0.78)	IL-1β	0.08 [0.07]	0.09 [0.07]	0.06 [0.08]
IL-1RN/ IL-1RA	11.05 (9.20)	12.10 (10.19)	13.68 (7.61)				
IL-2	13.33 (0.97)	13.29 (0.80)	13.22 (0.89)	IL-2	0.00 [0.00]	0.00 [0.00]	0.00 [0.00]
IL-4	11.54 (0.87)	10.90 (0.82)	10.59 (0.84)	IL-4	0.01 [0.05]	0.05 [0.06]	0.00 [0.04]
IL-5	13.59 (8.65)	13.68 (6.81)	13.87 (7.53)				
IL-6	12.52 (0.55)	11.56 (2.74)	12.48 (1.61)	IL-6	0.80 [0.31]	0.66 [0.62]	0.70 [0.86]
IL-7	9.69 (0.67)	9.53 (0.79)	9.50 (0.73)				
IL-9	17.35 (0.77)	16.74 (0.93)	15.71 (1.87)	IL-9	0.31 [0.23]	0.23 [0.27]	0.20 [0.014]
IL-10	13.67 (0.79)	13.95 (1.05)	13.66 (0.76)	IL-10	0.20 [0.16]	0.17 [0.13]	0.17 [0.12]
IL-12b	17.26 (0.87)	16.32 (1.42)	15.89 (2.74)	IL-12	0.11 [0.30]	0.00 [0.30]	0.00 [0.14]
IL-13	16.29 (1.37)	16.10 (1.17)	15.70 (0.98)				

IL-17a	16.67 (1.79)	15.53 (7.16)	14.04 (8.40)	IL-17A-F	0.00 [0.00]	0.00 [0.00]	0.00 [0.12]
IL-18	6.94 (1.07)	6.79 (1.77)	6.47 (2.02)				
IL-22	17.30 (1.15)	12.75 (8.96)	14.87 (6.83)				
IL-23	9.00 (0.43)	9.36 (0.57)	9.36 (0.53)				
IFN-γ	9.97 (0.62)	9.64 (0.84)	9.63 (0.76)	IFN-γ	6.69 [7.49]	6.65 [5.67]	6.21 [4.03]
TNF-α	6.57 (0.60)	6.37 (1.12)	6.48 (0.68)	TNF-α	0.56 [0.29]	0.50 [0.35]	0.53 [0.33]
CCL2	12.75 (1.33)	13.15 (1.08)	13.07 (0.97)	CCL2	263.16 [82.47]	288.87 [122.57]	299.20 [154.29]
CCL4	7.05 (0.73)	6.91 (0.88)	6.91 (0.61)				
CCL5	1.37 (0.56)	0.95 (0.66)	0.71 (0.56)	CCL5	93212.10 [72600.04]	140804.24 [100719.45]	134049.52 [107239.00]
CCL11	13.20 (0.00)	4.22 (11.78)	19.65 (0.00)				
CCL21	10.46 (12.65)	16.05 (7.05)	17.20 (4.12)				
CXCL5	6.85 (1.52)	5.97 (0.99)	5.42 (1.50)	CXCL5	1565.00 [1467.27]	1533.24 [1068.58]	1438.87 [991.59]
CXCL8	7.14 (1.57)	6.38 (0.91)	6.20 (0.87)	CXCL8	7.90 [4.14]	9.03 [5.13]	10.68 [4.08]
CXCL9	11.33 (1.01)	11.28 (0.96)	11.24 (0.90)				
CXCL10	10.33 (1.41)	10.83 (0.88)	10.59 (0.95)	CXCL10	190.02 [114.23]	233.83 [158.86]	231.69 [192.15]
CXCL11	11.63 (0.98)	11.76 (0.72)	11.79 (0.79)				

CX3CL1/ Fractalkine	17.33 (0.76)	14.77 (6.64)	16.71 (1.21)	CX3CL1	6061.66 [1333.26]	2099.72 [0.899]	6246.77 [1809.57]
CSF3/G-CSF	13.81 (8.77)	14.20 (7.59)	16.59 (2.73)	GM-CSF	0.00 [0.01]	0.00 [0.04]	0.00 [0.09]
VEGF-a	9.65 (0.46)	9.74 (0.53)	9.54 (0.53)	VEGF	74.75 [50.40]	94.88 [81.73]	103.40 [78.38]
NGF	4.99 (12.02)	8.83 (10.75)	12.32 (7.64)				
TGF-β1	2.75 (0.44)	2.53 (0.42)	2.38 (0.33)	TGF-β	42447.69 [15597.64]	55410.50 [20339.69]	54827.34 [20182.99]
PDGFA	9.93 (0.92)	9.54 (0.72)	9.27 (0.68)				
CRP	14.31 (7.67)	16.35 (2.05)	16.59 (1.64)	CRP	1.27 [2.70]	1.49 [1.54]	1.77 [1.96]
TAC1/ Substance P	-3.86 (10.76)	0.58 (12.13)	0.32 (14.23)				
PTGES2	6.83 (0.53)	7.55 (0.78)	7.50 (0.71)				
MMP9	5.08 (0.89)	4.72 (0.82)	4.47 (0.91)				
CD3D	4.33 (0.43)	4.41 (0.56)	4.58 (0.45)				
CD14	8.19 (0.55)	8.30 (0.58)	8.32 (0.59)				
FCGR3B/ CD16	7.43 (1.20)	6.44 (1.21)	5.99 (1.25)				
CD80	11.74 (0.58)	11.51 (0.86)	11.48 (0.70)				
CHI3L1	6.34 (1.71)	5.93 (1.18)	5.65 (1.15)				
TLR4	5.47 (0.79)	5.04 (0.64)	4.72 (0.92)				

NOS2	16.33 (1.29)	15.64 (2.45)	14.85 (4.32)

IQR = Inter quartile range, Ct = Cycle time.

Supplementary Table 5: Analysis of zero-inflated data using a zero-inflated hurdle model.

Odds ratio (exponentiated coefficients) of Hurdle model fitted to the Questionnaire data and IL9 gene expression (-log2 Normalised Cycle time) and IL4 protein expression (pg/ml). The model has two parts, a truncated Poisson model fitted on the non-zero scores and a Binomial model fitted on zero scores. Odds ratio < 1 in IL9 gene expression indicate higher expression. Odds ratio > 1 in IL4 protein expression indicate higher expression. The odds ratio for someone to have zero symptoms as measured by the NPSI total score, burning pain and paraesthesia subscores are increased with the expression of IL9 gene. The odds ration for someone to have higher positive (non-zero) scores in NPSI total and paraesthesia are increased for someone who has higher expression of IL4 protein expression.

Significance coding: p.value : < 0.001 ***, <0.01 **, <0.05 *

		Odds ratio	
		Count_model (Truncated Poisson)	Zero_hurdle_model (Binomial)
	Intercept	7.27	2.44
NPSI total	IL9_gene_Cycle_Time	0.93 ***	0.99
	Intercept	3.66 ***	1.56
NPSI burning	IL9_gene_Cycle_Time	0.98	1.1
NPSI	Intercept	4.23 ***	1.43
paraesthesia	IL9_gene_Cycle_Time	0.98	1
		Count_model (Truncated Poisson)	Zero_hurdle_model (Binomial)
	Intercept	20.45 ***	2.59 ***
NPSI total	IL_4_protein_expression_pg/ml	0.87 **	83.86
NPSI	Intercept	5.51 ***	0.86
paraesthesia	IL_4_protein_expression_pg/ml	0.93	4686.8 *

Supplementary Figure 1: Significant correlations of IL-9 levels and clinical phenotype. IL-9 protein levels negatively correlated with the NPSI composite score as well as the subdomains paraesthesia and paroxysmal pain. Pre- and post-surgery data were included in the analyses. Spearman's rank correlation was used with a $p<0.05$ being considered significant. A smoothed spline has been added to highlight the trend of the data. NPSI = Neuropathic pain symptom inventory

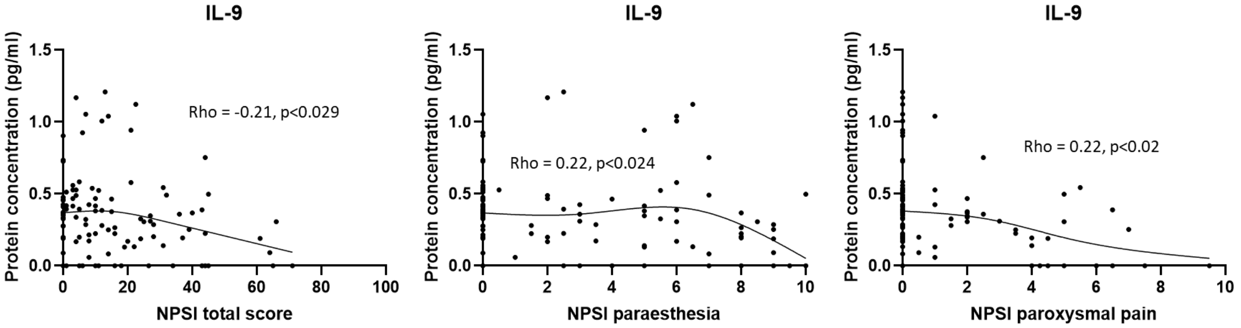

Supplement: Supplementary files [file EMS129569-supplement-Supplementary_files.pdf]
